# Benchmarking deep learning‐based low‐dose CT image denoising algorithms

**DOI:** 10.1002/mp.17379

**Published:** 2024-09-17

**Authors:** Elias Eulig, Björn Ommer, Marc Kachelrieß

**Affiliations:** ^1^ Division of X‐Ray Imaging and Computed Tomography German Cancer Research Center (DKFZ) Heidelberg Germany; ^2^ Faculty of Physics and Astronomy Heidelberg University Heidelberg Germany; ^3^ CompVis @ LMU Munich, MCML Munich Germany; ^4^ Medical Faculty Heidelberg Heidelberg University Heidelberg Germany

**Keywords:** benchmarking, computed tomography, deep learning, denoising, low‐dose

## Abstract

**Background:**

Long‐lasting efforts have been made to reduce radiation dose and thus the potential radiation risk to the patient for computed tomography (CT) acquisitions without severe deterioration of image quality. To this end, various techniques have been employed over the years including iterative reconstruction methods and noise reduction algorithms.

**Purpose:**

Recently, deep learning‐based methods for noise reduction became increasingly popular and a multitude of papers claim ever improving performance both quantitatively and qualitatively. However, the lack of a standardized benchmark setup and inconsistencies in experimental design across studies hinder the verifiability and reproducibility of reported results.

**Methods:**

In this study, we propose a benchmark setup to overcome those flaws and improve reproducibility and verifiability of experimental results in the field. We perform a comprehensive and fair evaluation of several state‐of‐the‐art methods using this standardized setup.

**Results:**

Our evaluation reveals that most deep learning‐based methods show statistically similar performance, and improvements over the past years have been marginal at best.

**Conclusions:**

This study highlights the need for a more rigorous and fair evaluation of novel deep learning‐based methods for low‐dose CT image denoising. Our benchmark setup is a first and important step towards this direction and can be used by future researchers to evaluate their algorithms.

## 1 INTRODUCTION

Computed tomography (CT) is an important imaging modality, with numerous applications including biology, medicine, and nondestructive testing. However, the use of ionizing radiation remains a key concern and thus clinical CT scans must follow the ALARA (as low as reasonably achievable) principle.^1^, ^2^ Therefore, reducing the dose and thus radiation risk is of utmost importance and one of the primary research areas in the field.

A straightforward approach to reduce dose is by lowering the tube current (i.e., reducing the x‐ray intensity). However, this comes at the cost of deteriorated image quality due to increased image noise and thus potentially reduced diagnostic value. To alleviate this drawback, numerous algorithms have been proposed to solve the task of low‐dose CT (LDCT) denoising, that is, reducing image noise in the reconstructed image (or volume).

Iterative reconstruction (IR) techniques incorporate prior knowledge in the reconstruction process and then update the reconstructed image iteratively. The prior knowledge may model statistical properties of the noise,^3^ properties of the object to be reconstructed,^4^ or parameters of the CT system. While IR techniques can be used to reduce numerous other artifacts compared to conventional filtered back projection (FBP), they are computationally expensive, which limits their clinical applicability. On the other hand, filtering techniques to reduce noise are fast and easy to implement into various reconstruction frameworks. The filtering may either be performed in projection domain, image domain, or both, and using a wide range of algorithms.^5^, ^6^, ^7^, ^8^, ^9^ Recently deep learning‐based filtering, particularly in the image domain, became increasingly popular.^10^, ^11^, ^12^, ^13^, ^14^, ^15^, ^16^, ^17^, ^18^, ^19^, ^20^, ^21^, ^22^ The majority of the proposed methods learn a mapping from low‐dose images to high‐dose images in a supervised fashion using a deep neural network (DNN). Of the numerous proposed methods, most suggestions for improvement alter the network structure, loss function, or training strategy. Publications often claim ever improving performance which is commonly demonstrated by improved image quality metrics (e.g., peak signal‐to‐noise ratio, structural similarity) in experiments on simulated or clinical data.

In this work, we identify several flaws in the experimental setup of such methods which limit the verifiability of the claimed improvements. These include the lack of a common benchmark dataset, the use of inadequate metrics with little relation to diagnostic value, and unfair choice of hyperparameters for reference methods. Reproducibility and verifiability of scientific results, however, are paramount to scientific advancements of a field, and thus efforts towards fair benchmarking of existing and future algorithms are of utmost importance. To this end, we make the following contributions:

1. We identify multiple flaws in the experimental setup of previously proposed methods which hinder the verifiability of their claimed improvements.
2. We propose a benchmark setup^1^ for deep learning‐based LDCT denoising methods, which aims to overcome those flaws and allows for a fair evaluation of existing algorithms and those yet to come.
3. In a comprehensive and fair evaluation of several existing algorithms we find that there has been little progress over the past six years and many of the newer methods perform statistically similar or worse compared to older ones.

## 2 RELATED WORK

In this section, we review existing works on deep learning‐based LDCT denoising and image quality assessment (IQA) of medical images.

### 2.1 Deep learning‐based LDCT denoising

CT image reconstruction aims at solving the linear system Rx=p, with $p\in R^{M}$ denoting the measurements in projection domain, $x\in R^{N}$ being the volume to be reconstructed, and $R\in R^{M\times N}$ the Radon transform. LDCT generally aims at reconstructing x using less dose, which can be for example, accomplished by lowering the tube current, thus increasing the noise in p and x, or by lowering the number of measurements M, leading to sparse‐view artifacts in x. Since previous studies indicate that DNN‐based correction of the former can be superior, we here consider the task of LDCT denoising.^23^ Based on the domain (p,x, or both) in which they operate, deep learning‐based methods for LDCT image denoising can be divided into three categories: projection‐domain, image‐domain, and dual‐domain.

Projection‐domain methods aim to learn a mapping $f_{\theta }:p^{\prime }\to p$ from low‐dose projections $p^{\prime }$ to high‐dose projections p, where $f_{\theta^{*}}$ is realized by a DNN, parameterized by weights θ. These weights are either optimized in a supervised setting via

(1) $$\begin{array}{c} \theta^{*}=\underset{\theta }{\arg\min}\;E_{p^{\prime },p∼D^{\text{train}}}∥f_{\theta }\left(p^{\prime }\right)-p∥\;, \end{array}$$

with ∥·∥ being some norm,^24^, ^25^ or unsupervised, exploiting structural similarities between adjacent projections.^26^, ^27^ The denoised projections can then be reconstructed using either of the standard reconstruction techniques.^28^, ^29^, ^30^

Image‐domain methods aim to directly learn a mapping $g_{\phi }:x^{\prime }\to x$ from low‐dose images $x^{\prime }$ (i.e., images reconstructed from low‐dose projections $p^{\prime }$ using FBP) to high‐dose images x. Similar to Equation (1), weights are typically optimized in a supervised setting, where the mean‐squared error (MSE), or some other pixel‐ or feature‐based loss between prediction and high‐dose image x is minimized,^10^, ^11^, ^12^, ^13^, ^14^, ^17^, ^19^, ^21^, ^22^, ^31^ or g is trained together with a discriminator as a generative adversarial network (GAN).^15^, ^18^, ^20^ Notable other works investigate unsupervised‐ or self‐supervised training strategies, or leverage the intrinsic image prior of DNNs.^32^

Lastly, dual‐domain methods operate in both domains x and p simultaneously, by employing two separate networks f and g, respectively. Networks are trained either separately using aforementioned loss functions^33^, ^34^ or in an end‐to‐end fashion using a differentiable analytical reconstruction layer.^35^, ^36^, ^37^

In this work we focus on image‐domain methods which dominate the research field. This is mainly due to the abundance of open source datasets, where paired high‐ and low‐dose images are readily available.^38^, ^39^ In contrast, projection data are generally proprietary and thus difficult to access.^40^ The few datasets that provide them usually do so only for a (vendor‐specific) subset of the data and handling of them can be cumbersome due to (hidden) preprocessing steps in the reconstruction pipeline of the vendor.^39^, ^41^ Many of the principles in the design of our benchmark setup, however, can be applied to the evaluation of projection‐domain or dual‐domain methods as well.

### 2.2 Medical image quality assessment

Common full‐reference quantitative measures for natural image quality assessment include the structural similarity index measure (SSIM)^42^ and peak signal‐to‐noise ratio (PSNR). However, these metrics are usually not in agreement with human readers, which are considered the gold standard for image quality assessment of medical images.^43^, ^44^, ^45^ These are conducted by measuring the accuracy of multiple radiologists when performing some task (e.g., lesion detection or segmentation) using certain images. However, this metric relies, and is dependent on the definition of a suitable task. Therefore, the subjective assessment of overall diagnostic quality by radiologists is a common alternative measure.^46^ Nonetheless, since conducting multiple‐reader studies is time‐consuming and expensive, most algorithms for enhancement of medical images are still evaluated using quantitative metrics such as SSIM or PSNR.

In refs. [45, 46], the authors find that multiple other metrics, including the visual information fidelity (VIF),^47^ have higher correlation with human reader ratings compared to SSIM and PSNR for both CT and magnetic resonance (MR) images. Furthermore, notable recent works investigate the use of radiomic features to provide a clinically meaningful measure for the quality of medical images without the drawbacks of human reader studies.^48^, ^49^, ^50^

Moreover, many physical image quality metrics for evaluating different aspects of the technical performance of CT equipment exist such as the modulation transfer function (MTF), contrast‐to‐noise ratio (CNR), noise power spectrum (NPS), and CT number accuracy. However, these quantities often rest on strong assumptions about the imaging system and reconstruction algorithm such as linearity, shift‐invariance, or stationarity of the noise, many of which are violated for IR or deep learning‐based reconstruction methods.^51^, ^52^, ^53^, ^54^ Another drawback is that these metrics are commonly evaluated using phantom measurements, thus posing an out‐of‐distribution problem for deep learning‐based methods which are trained exclusively on clinical data. Even for methods that are trained on a mixture of phantom and patient data (e.g., GE Healthcare's TrueFidelity™^55^), results from phantom measurements may not be representative of the performance on clinical data.

## 3 FLAWS OF CURRENT EVALUATION PROTOCOLS

**FIGURE 1:**
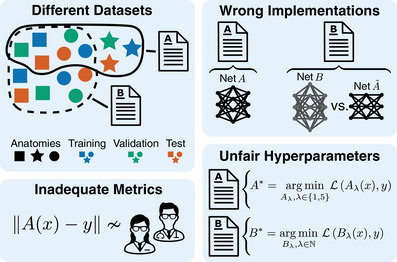
Overview of flaws in the experimental setup of many deep learning‐based LDCT denoising methods, that limit their verifiability.

In this section we will outline the main problems with current evaluation protocols for deep learning‐based image‐domain LDCT denoising (see Figure 1 for an overview).

### 3.1 Different datasets

Unlike in many other disciplines of computer vision, particularly image denoising of natural images,^56^, ^57^, ^58^, ^59^, ^60^ there exist no consensus regarding benchmark datasets for LDCT denoising. While most methods are trained and evaluated on the dataset provided as part of the *2016 NIHAAPM‐Mayo Clinic LDCT Grand Challenge* ^38^ or the subsequently released (significantly larger both in number of images and anatomical sites) *LDCT and Projection data*,^39^ authors of each method employ their own training, validation, and test split. Therefore, reported metrics across publications are not comparable. This is further exacerbated by the fact that performance of individual methods differs significantly between different anatomical sites and images (i.e., axial slices), as shown by our experiments.

### 3.2 Unfair choice of hyperparameters

Very few publications on LDCT denoising methods report the application of hyperparameter optimization^61^, ^62^, ^63^ for their own or the considered comparison methods. In none of the respective publications of the algorithms considered in this study, exhaustive hyperparameter optimization is performed. The 3/8 algorithms that report some form of hyperparameter optimization limit it to a grid search with few points over a single parameter (learning rate),^13^, ^19^ a subset of the comparison methods,^13^ or their own method.^15^ Often, authors simply use the hyperparameters reported in the reference publications.^12^, ^13^, ^15^ This is particularly problematic given the choice of different datasets (cf. Section 3.1), where hyperparameters optimized by authors of method A on dataset $D_{A}$ may not be optimal for the dataset $D_{B}$ employed by authors B in their experiments.

### 3.3 Missing open source implementations

With many authors not providing open source implementations of their algorithms, researchers are often left to implement comparison methods themselves. This increases the chances of errors.^64^ Additionally, changing other aspects (such as the architecture of comparison methods^13^) can further bias experimental results.

### 3.4 Inadequate metrics

Most LDCT denoising methods are evaluated using SSIM,^42^ PSNR, or root‐mean‐square error (RMSE). While these are common metrics to quantify performance for natural image denoising, they are usually not in agreement with human readers for medical images (cf. Section 2.2), making it difficult to assess the extent to which the reported improvements actually translate into clinical benefits. This could be improved by the use of quantitative measures that are more suited for medical images (e.g., VIF), or experiments using human reader studies. In the respective publications of the eight algorithms considered in this study, however, most are evaluated using SSIM, RMSE, and PSNR only. Better metrics such as VIF or reader studies are employed in three publications only.

## 4 BENCHMARK SETUP

In the following we present a benchmark setup to overcome the flaws of current evaluation protocols, as outlined in Section 3 that allows for a fair and clinically meaningful evaluation of DNNs for LDCT denoising.

### 4.1 Dataset

For our benchmark setup we utilize the publicly available *Low dose CT and Projection Dataset*,^39^ comprising a total of 150 scans of abdomen, head, and chest, (50 scans for each exam type) at routine dose levels. For each scan, simulated low‐dose reconstructions (by means of noise insertion in the projection domain) at 25% dose for abdomen/head and 10% dose for chest, are available. For each exam type separately, data are split in 70%/20%/10% training/validation/test set and then linearly normalized to have zero mean, unit variance. Future studies might consider treating data normalization as an additional hyperparameter.^65^ During training, we employ a weighted sampling scheme such that slices from each exam type and patient are sampled with equal probability. During testing, we reduce each scan to axial regions where the brain is present (for head scans), the lung is present (for chest scans), or the lung is not present (abdomen). We did not apply any data augmentation to the training data as we did not observe overfitting in our experiments for any of the methods. The code to reproduce exact dataset splits and all preprocessing is included in our benchmark suite.

### 4.2 LDCT denoising algorithms

We consider eight DNN‐based LDCT denoising algorithms proposed in the literature over the past six years. In the following we briefly describe each of the methods and refer the reader to the respective publications for more details. **CNN‐10** ^10^ is a simple three layer CNN, trained to minimize the MSE between network output and high‐dose targets. **RED‐CNN** ^12^ and **ResNet** ^31^ are trained in the same fashion but employ deeper network architectures with residual connections compared to CNN‐10. **WGAN‐VGG** ^20^ and **DU‐GAN** ^15^ are trained in an adversarial fashion,^66^, ^67^ where DU‐GAN utilizes a U‐net based discriminator.^68^ **QAE** ^13^ is based on RED‐CNN in both network architecture and training scheme, but employs quadratic convolutions. **TransCT** ^22^ is based on transformer blocks and also trained with an MSE loss. **Bilateral** ^19^ uses a trainable bilateral filter instead of a DNN, and thus substantially reduces the amount of free model parameters.

In Appendix A, we provide details on our implementation and verification of each of the algorithms.

### 4.3 Hyperparameter optimization

As discussed in Section 3.2, for none of the methods a rigorous hyperparameter optimization was employed in the original publications. To ensure a fair comparison between different algorithms we optimize hyperparameters as follows. For each method we first identify hyperparameters and their suitable ranges. This includes general parameters such as learning rate, mini‐batch size, patchsize and number of iterations, but also weighting factors in the loss functions (e.g., to balance adversarial and pixelwise loss in a GAN setting). Suitable ranges were determined from the respective papers (with sufficient margin) and whenever two methods had the same hyperparameter (e.g., learning rate or patchsize), we kept the prior distribution over the search space the same. All hyperparameters and their respective prior distributions are reported in Table 1. For each method, we then performed a black box hyperparameter tuning using sequential‐model based optimization (SMBO). Such an automatic approach is preferred over manual (human) optimization as it avoids any potential bias by the practitioner, thus ensuring fair comparison of different models. Furthermore, SMBO has been shown to outperform both human optimization and non‐sequential optimization schemes like grid search or random search on a variety of DNN and dataset combinations.^61^, ^63^

**TABLE 1:** Hyperparameters for all deep‐learning based LDCT denoising methods considered in this study.

	Parameter	Prior
All algorithms	Learning rate	$\log U(1\times 10^{-5},0.01)$
	Maximum iterations	$U(1\times 10^{3},1\times 10^{5})$
	Mini‐batch size	U(2,128)
CNN‐10^10^	Patchsize	U(32,128)
RED‐CNN^12^	Patchsize	U(32,128)
WGAN‐VGG^20^	$\beta_{1}$ of Adam	U(0.3,0.9)
	Loss weight: $\lambda_{\text{perceptual}}$	U(0,1)
	Critic updates	U(1,5)
	Patchsize	U(32,128)
ResNet^31^	Patchsize	U(32,128)
QAE^13^	Patchsize	U(32,128)
DU‐GAN^15^	$\beta_{1}$ of Adam	U(0.3,0.9)
	Cutmix warmup	$U(0,1\times 10^{4})$
	Loss weight: $\lambda_{\text{adv}}$	U(0,1)
	Loss weight: $\lambda_{\text{CM}}$	U(0,10)
	Loss weight: $\lambda_{\text{px,grad}}$	U(0,40)
	Critic updates	U(1,5)
	Patchsize	U(32,128)
TransCT^22^	—	—
Bilateral^19^	Learning rate for $\sigma_{r}$	$\log U(1\times 10^{-5},0.01)$
	Patchsize	U(32,128)
	Initalization for $\sigma_{r}$	U(0,1)
	Initalization for $\sigma_{x,y}$	U(0,1)

*Note*: The first three parameters are optimized for all algorithms (separately).

Abbreviations: U: uniform distribution; logU: log‐uniform distribution.

Let $t_{\lambda }:\left\{f_{\theta },D^{\text{train}},\lambda \right\}\to \theta^{*}$ denote the outcome of some training run of network f on training data $D^{\text{train}}$ using hyperparameters λ. The aim of hyperparameter optimization is to find an optimial set of hyperparameters $\lambda^{*}$, that is,

(2) $$\begin{array}{cc} \lambda^{*} & =\underset{\lambda }{\arg\max}\;E_{x,y∼D^{\text{val}}}\left[M\left(y,f_{t_{\lambda }}\left(x\right)\right)\right]\\ & =\underset{\lambda }{\arg\max}\;\Psi \left(\lambda \right)\;, \end{array}$$

where M is some metric and $D^{\text{val}}$ the validation dataset (not used during $t_{\lambda }$). Since evaluating Ψ(λ) is expensive, requiring a full training run $t_{\lambda }$, one uses a probabilistic model $p_{\Psi }$, here constructed via Gaussian processes, as a surrogate for Ψ. For each iteration in the optimization process, we then find the most promising next point λ, to run the costly evaluation Ψ(λ) for, by maximizing some acquisition function. In our experiments we used the expected improvement (EI)^61^ as acquisition function:

(3) $$\begin{array}{c} \text{EI}\left(\lambda ,\Psi^{*}\right)=\int \max\left(z-\Psi^{*},0\right)\;p_{\Psi }\left(z|\lambda \right)dz\;, \end{array}$$

where $\Psi^{*}$ refers to the expectation of M on the validation data for the best set of hyperparameters found so far (i.e., the one that maximizes the r.h.s. of Equation 2 up to now). As metric M that is optimized by the hyperparameter optimization, we used the SSIM for all networks. Optimizing the SSIM is favorable over other measures, since it is fast to compute, unlike for example, VIF, and not directly involved in the training process $t_{\lambda }$ of any of the methods considered in this study (unlike e.g., RMSE). Further, note that for methods using a vanilla GAN loss, for example, ref. [15], simply minimizing the validation loss would not be suitable as it is not directly related to training progress. For each method, we perform 50 iterations of SMBO, sufficient to ensure convergence for all algorithms, as verified by our experiments.

After an optimal set of hyperparameters $\lambda^{*}$ was found, we retrained a method using $\lambda^{*}$ 10 times with different random seeds. If not stated otherwise, all reported standard deviations and significance tests (to compare two methods) are computed over those 10 training runs.

### 4.4 Metrics

We evaluate all methods on the same test set comprising a total of 15 scans (5 head/chest/abdomen) using three common full‐reference measures of image quality: SSIM, PSNR^2^, and VIF. As described in Section 3.4, both SSIM and PSNR are common metrics to evaluate DNNs for LDCT denoising. We include VIF, since it has been shown to have higher correlation with human readers for medical images.^45^, ^46^

Conducting human reader studies is time‐consuming and expensive and would render the application of the proposed benchmark setup to future algorithms impossible. To nevertheless evaluate the algorithms in terms of clinically relevant image properties, we include an analysis of radiomic features. To this end, we compare the similarity of radiomic features extracted on the denoised images to those extracted on the high‐dose image.

Definition 1 (Radiomic feature similarity) Let cos(x,y) be the cosine similarity between two vectors x and y:

(4) $$\begin{array}{c} \cos\left(x,y\right)=\frac{x\cdot y}{∥x∥∥y∥}\;. \end{array}$$

Further, let A={0,1,2,⋯,n}, with n being the number of algorithms considered, and index 0 being associated with the high‐dose image. We denote with $R_{i,j}^{\left(s\right)}$ the radiomic feature j∈{1,2,⋯,J} extracted on scan s associated with algorithm i∈A. In order to get a task‐agnostic metric, we assign an equal a‐priori importance to each feature by normalizing

(5) $$\begin{array}{c} {\tilde{R}}_{i,j}^{\left(s\right)}=\frac{R_{i,j}^{\left(s\right)}-\max_{k\in A}R_{k,j}^{\left(s\right)}}{\max_{k\in A}R_{k,j}^{\left(s\right)}-\min_{k\in A}R_{k,j}^{\left(s\right)}}\;. \end{array}$$

The radiomic feature similarity ${\text{RFS}}_{i}^{\left(s\right)}$ of algorithm i=1,…,n on some scan s is then given as

(6) $$\begin{array}{c} {\text{RFS}}_{i}^{\left(s\right)}=\cos\left(r_{i}^{\left(s\right)},r_{0}^{\left(s\right)}\right),\;r_{i}^{\left(s\right)}=\left({\tilde{R}}_{i,1}^{\left(s\right)},\cdots {\tilde{R}}_{i,J}^{\left(s\right)}\right)\;. \end{array}$$

Radiomic features are commonly extracted on segmentations of tumors or entire organs. On the high‐dose scans of the test data, we therefore segment the following organs using the TotalSegmentator^69^: lung on chest scans, liver on abdomen scans, and brain on head scans. This segmentation mask is then used for subsequent extraction of 91 radiomic features^3^ using PyRadiomics.^70^ Note, that because the same segmentation (on high‐dose scans) is used for all algorithms, shape‐based features (e.g., voxel volume) were excluded for computation of the RFS.

Furthermore, we evaluate the algorithms in terms of their ability to reconstruct lesions and using classical image quality metrics for CT (Sections 5.5 and 5.6).

### 4.5 LDCT‐hard benchmark dataset

In our experiments we find that the performance of all algorithms varies greatly, both between different exam types and images of the same exam type. The latter observation motivates us to derive a novel collection of test datasets, each of which being a subset of the *Low‐dose CT and Projection Dataset*.^39^ We refer to LDCT‐hard‐q%, as the subset containing the q% slices with lowest average SSIM across all evaluated methods. To not underrepresent anatomies for which methods achieve generally higher SSIMs (e.g., head), this subset is collected for each exam type separately.

## 5 RESULTS

### 5.1 Hyperparameter optimization

**FIGURE 2:**
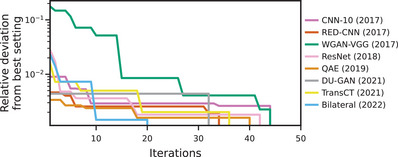
Evolution of relative deviation from best setting over the 50 iterations of Bayesian hyperparameter optimization. For each iteration i we show the relative deviation of the best network up to i from the final best configuration of hyperparameters (over all 50 iterations).

We first verify that all methods converged within the 50 iterations of Bayesian hyperparameter optimization (Figure 2). To this end, we evaluate for each method and iteration i the relative deviation ${\text{RelDev}}_{i}$ from the best setting w.r.t. the SSIM on the validation set

(7) $$\begin{array}{c} {\text{RelDev}}_{i}=1-\frac{\max_{j\leq i}{\text{SSIM}}_{j}}{\max_{j}{\text{SSIM}}_{j}}\;. \end{array}$$

We find that hyperparameter optimization for most of the methods converged within the first 40 iterations and none of the methods improved in the last five iterations (cf. intercept with *x*‐axis in Figure 2). For all methods ${\text{RelDev}}_{i\geq 30}<0.5\%$.

### 5.2 Evaluation using standard image quality metrics

We then evaluate all algorithms using the following image quality metrics: SSIM, PSNR, and VIF (Table 2). For each method, we test if it performs significantly better or worse than the previously published best method, using the nonparametric *Mann‐Whitney U* test^71^ with significance level α=5%. While we find that ResNet significantly outperforms previous methods on the chest data, none of the newer methods consistently outperforms RED‐CNN, one of the earliest deep learning‐based methods for LDCT denoising (cf. **bold** numbers in Table 2). On the contrary, for many configurations newer methods perform significantly worse than RED‐CNN (cf. *italic* numbers in Table 2). In particular, we find that the two newest methods considered in this study (TransCT and Bilateral) perform significantly worse w.r.t. all metrics and exam types compared to RED‐CNN. Remarkably, they even perform significantly worse compared to the low‐dose scan on few metric and exam type combinations (e.g., TransCT on head scans for all metrics; Bilateral on abdomen scans for PSNR).

**TABLE 2:** Quantitative evaluation using the metrics SSIM, PSNR, and VIF.

	Chest (10% dose)			Abdomen (25% dose)			Head (25% dose)			
	SSIM	PSNR (dB)	VIF	SSIM	PSNR (dB)	VIF	SSIM	PSNR (dB)	VIF	Rank
LD	0.34	18.77	0.09	0.84	28.67	0.34	0.88	26.4	0.55	9
CNN‐10	**0.5867** ± **0.0006**	**27.71** ± **0.02**	**0.1915** ± **0.0008**	**0.896** ± **0.001**	**32.4** ± **0.1**	**0.449** ± **0.003**	**0.896** ± **0.004**	**28.9** ± **0.6**	**0.620** ± **0.006**	3
RED‐CNN	**0.609** ± **0.002**	**28.36** ± **0.03**	**0.221** ± **0.003**	**0.9028** ± **0.0007**	**33.22** ± **0.07**	**0.491** ± **0.008**	**0.904** ± **0.001**	**30.4** ± **0.2**	**0.69** ± **0.01**	1
WGAN‐VGG	*0.51* ± *0.03*	*25.5* ± *0.2*	*0.148* ± *0.004*	*0.882* ± *0.002*	*30.5* ± *0.9*	*0.38* ± *0.01*	*0.88* ± *0.02*	*25* ± *3*	*0.53* ± *0.02*	6^†^
ResNet	**0.610** ± **0.001**	**28.42** ± **0.03**	**0.224** ± **0.002**	*0.901* ± *0.002*	*33.15* ± *0.08*	*0.487* ± *0.006*	*0.901* ± *0.005*	*29.6* ± *0.8*	*0.67* ± *0.02*	2
QAE	*0.584* ± *0.003*	*27.62* ± *0.09*	*0.186* ± *0.003*	*0.894* ± *0.002*	*32.0* ± *0.2*	*0.418* ± *0.007*	*0.899* ± *0.001*	*28.5* ± *0.3*	*0.594* ± *0.008*	5
DU‐GAN	*0.565* ± *0.004*	*26.7* ± *0.1*	*0.168* ± *0.002*	*0.894* ± *0.002*	*32.1* ± *0.3*	*0.427* ± *0.005*	0.903 ± 0.003	*29* ± *1*	*0.622* ± *0.005*	4
TransCT	*0.563* ± *0.002*	*26.99* ± *0.05*	*0.167* ± *0.002*	*0.877* ± *0.003*	*30.5* ± *0.2*	*0.372* ± *0.007*	*0.849* ± *0.005*	*24.7* ± *0.4*	*0.44* ± *0.01*	6^†^
Bilateral	*0.555* ± *0.001*	*25.59* ± *0.04*	*0.159* ± *0.002*	*0.859* ± *0.003*	*27.1* ± *0.1*	*0.361* ± *0.003*	*0.873* ± *0.002*	*26.6* ± *0.1*	*0.500* ± *0.004*	8

*Note*: We highlighted a metric in **bold**, if it is significantly better than the previously published best method on that anatomy. Likewise, we highlighted a metric in *italics*, if it is significantly worse than the previously published best method on that anatomy. The rank column (last column) is the competition ranking over all anatomies and metrics. We indicate a tie with ^†^.

### 5.3 Evaluation using radiomic feature similarity

We further evaluate all algorithms using the radiomic feature similarity in order to better assess whether the differences observed in the previous section translate to clinical features.

**FIGURE 3:**
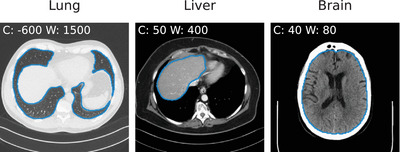
Contour plots of automatic segmentations for three high‐dose scans of the test set of lung, liver, and brain. Radiomic features were extracted within these segmentations for low‐ and high‐dose as well as all denoised volumes.

In Figure 3 we show contour plots of the automatic segmentations of the brain, lung, and liver for three high‐dose scans of the test set. We visually verify that segmentations are reasonably good for all 15 scans in the test set. Those segmentation masks are then used to extract radiomic features for all low‐ and high‐dose, as well as all denoised volumes of the test set. Using the same segmentation mask for subsequent radiomic feature extraction of all algorithms ensures a fair comparison, despite possible small errors produced by the automatic segmentation pipeline.

**FIGURE 4:**
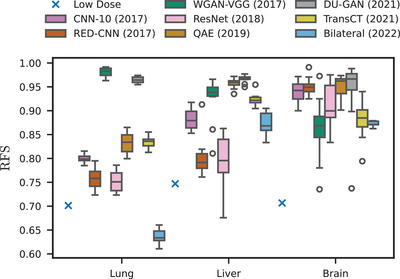
Radiomic feature similarity for different exam types and methods. Individual samples correspond to mean RFS over all scans of an anatomy for a single trained network. Box plots were then drawn over the 10 training runs with different random seeds (cf. Section 4.3.).

**FIGURE 5:**
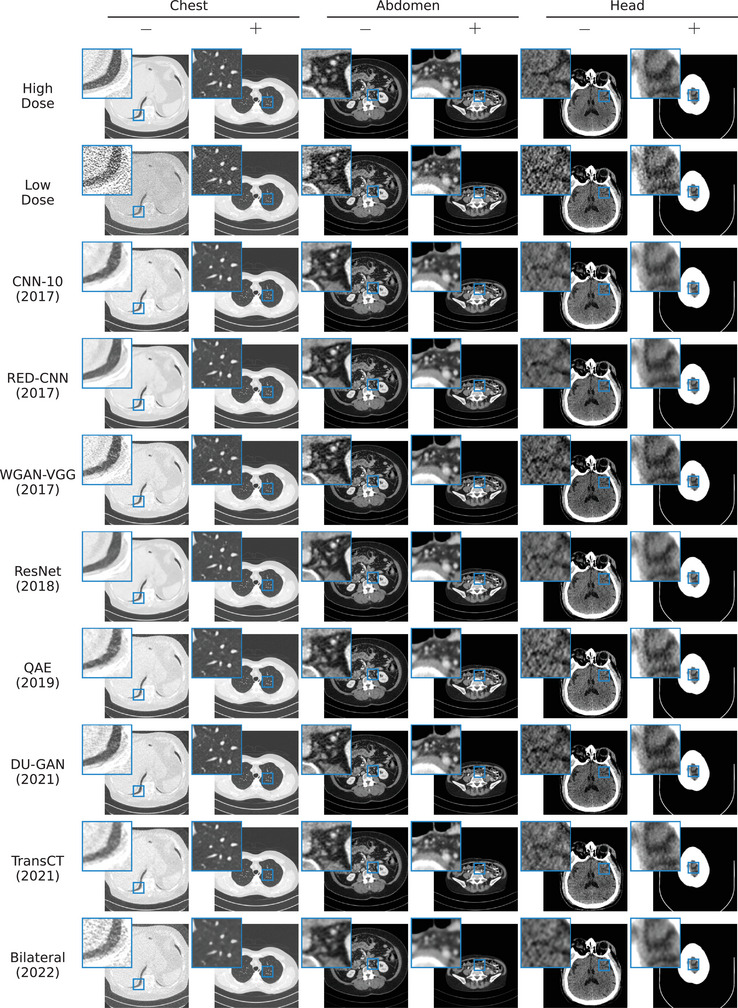
Best viewed zoomed in. Slices from the test dataset, for which the average SSIM over all methods is lowest (−) and highest (+). For each method, we show results for the best performing network (over the 10 random trials), that is, network having the highest SSIM on the validation data.

Upon evaluation of the radiomic feature similarity (Table 3 and Figure 4), we find that radiomic features extracted for all denoising methods are significantly more similar to those extracted on the high‐dose scan, compared to features extracted on the low‐dose scan, with Bilateral on lung data being the only exception. We also find that contrary to our findings using standard image quality metrics, RED‐CNN is outperformed by numerous other algorithms, including the (older) CNN‐10, and newer algorithms such as WGAN‐VGG and QAE. Remarkably, the two GAN‐based algorithms WGAN‐VGG and DUGAN outperform all other algorithms on the lung data by a large margin. We hypothesize that this is due to the lower dose (10% vs. 25% for all other anatomies) on that data and the ability of GANs to produce more realistic noise textures in high‐ambiguity settings compared to methods trained with standard pixelwise loss functions.^72^ Nonetheless, we do not find newer algorithms to consistently outperform older ones, and particularly the two newest algorithms considered in our study (TransCT and Bilateral) perform significantly worse w.r.t. radiomic feature similarity of all organs compared to older methods. Figure 5 shows qualitative results for the slices from the test dataset for which the average SSIM over all methods is lowest (−) and highest (+), respectively. As can be seen, for each anatomy, the slice maximizing the average SSIM is the one where the cross sectional area of the patient is small, thus reducing the noise in the low‐dose image.

**TABLE 3:** Quantitative evaluation using the radiomic feature similarity.

	Lung	Liver	Brain	Rank
LD	0.7	0.75	0.71	9
CNN‐10 (2017)	**0.800** ± **0.009**	**0.88** ± **0.02**	**0.94** ± **0.02**	4^†^
RED‐CNN (2017)	*0.76* ± *0.02*	*0.80* ± *0.04*	0.95 ± 0.02	6
WGAN‐VGG (2017)	**0.98** ± **0.01**	**0.92** ± **0.05**	*0.86* ± *0.07*	4^†^
ResNet (2018)	*0.75* ± *0.02*	*0.79* ± *0.06*	0.91 ± 0.05	7
QAE (2019)	*0.83* ± *0.02*	**0.96** ± **0.01**	0.95 ± 0.02	2
DU‐GAN (2021)	*0.965* ± *0.007*	**0.967** ± **0.008**	0.94 ± 0.08	1
TransCT (2021)	*0.83* ± *0.01*	*0.92* ± *0.01*	*0.88* ± *0.04*	3
Bilateral (2022)	*0.64* ± *0.01*	*0.87* ± *0.02*	*0.873* ± *0.006*	8

*Note*: **Bold** numbers indicate that a method is significantly better than the previously published best method on that anatomy. Likewise, italics indicate that it is significantly worse. The rank column (last column) is the competition ranking over all anatomies and metrics. We indicate a tie with ^†^.

### 5.4 Evaluation on LDCT‐hard datasets

**FIGURE 6:**
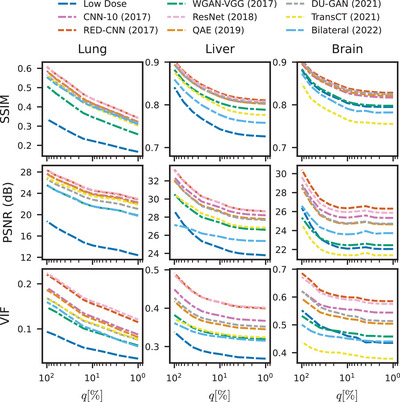
Evaluation of all methods for LDCT‐hard‐*q*% for different values of *q* (right is smaller). For some settings and anatomies, methods perform up to 50% worse for small *q*. The regular test set corresponds to *q* = 100%. Errorbars were omitted to improve visibility.

Figure 6 shows the performance of individual methods for increasingly hard subsets of the training data (i.e., smaller q). We find a strong correlation between metrics for each method and the low‐dose scan. Although not surprising, this indicates that methods perform increasingly worse for increasing deviations of the low‐dose scan from the high‐dose scan. Additionally, the ranking among methods remains mostly invariant to q, and thus we conclude that all methods are similar in terms of their robustness to different amounts of deterioration of the low‐dose scan. Remarkably, WGAN‐VGG, having a lower VIF and PSNR compared to the low‐dose scan on head exams for the regular test set (corresponding to q=100%), has a higher VIF and PSNR compared to the low‐dose scan for more difficult slices (q≤16% for VIF, q≤40% for PSNR). This may be explained by the aforementioned ability of GANs to produce more realistic results in high‐ambiguity settings compared to networks trained in a pixelwise fashion.

### 5.5 Evaluation on lesions

The main downstream task for clinical low‐dose CT is the detection and diagnosis of lesions. To better assess whether the denoising algorithms improve performance on this downstream task compared to low‐dose reconstructions we utilize the lesion annotations provided with the *LDCT Image and Projection dataset*.^39^ For our test set there exist a total of eleven annotations covering all three exam types and six different diagnosis. For each of these lesions we compute the RMSE and PSNR compared to the high‐dose reconstruction within the bounding‐box surrounding the lesion (Table 4).

**TABLE 4:** Quantitative evaluation of the algorithms ability to reconstruct lesions for the three anatomies, averaged over lesions.

	Chest (10% dose)		Abdomen (25% dose)		Head (25% dose)		Rank
	PSNR (dB)	RMSE (HU)	PSNR (dB)	RMSE (HU)	PSNR (dB)	RMSE (HU)	
LD	9.67	169.21	12.39	23.68	9.76	8.43	9
CNN‐10 (2017)	13.2	73.24	14.59	14.23	11.89	5.15	3
RED‐CNN (2017)	**13.36**	**71.98**	**14.87**	**13.39**	**12.28**	**4.74**	1
WGAN‐VGG (2017)	12.11	94.79	14.02	16.41	10.65	6.85	8
ResNet (2018)	13.28	73.15	14.82	13.53	12.1	4.92	2
QAE (2019)	12.94	77.93	14.2	15.54	11.28	5.93	6^†^
DU‐GAN (2021)	13.04	76.27	14.46	14.73	11.06	6.23	5
TransCT (2021)	13.04	82.57	14.41	14.94	11.12	6.12	6^†^
Bilateral (2022)	12.6	84.53	14.72	13.92	12.05	4.98	4

*Note*: We indicate the best performing method for an anatomy and metric in **bold**. The rank column (last column) is the competition ranking over all anatomies and metrics. We indicate a tie with^†^.

Here, we find that all methods have lower deviations from the ground truth compared to the low‐dose reconstruction. We also find that the ranking mostly agrees with the ranking based on standard image quality metrics (cf. Table 2) and in particular w.r.t. the three best performing methods (RED‐CNN > ResNet > CNN‐10) both rankings agree. We provide reconstruction results for all lesions and algorithms in the Appendix, Figures C.4 to C.6.

### 5.6 Evaluation using physical CT IQA metrics

We also analyzed the algorithms using physical image quality metrics, a common way to evaluate the technical performance of CT systems. A discussion on the limitations of these metrics is provided in Section 2.2. In particular, we perform all evaluations using patient scans of the test set instead of phantom measurements to avoid an out‐of‐distribution setting. In the following, we provide the main results of this evaluation and refer to Appendix D for additional results.

#### Contrast‐to‐noise ratio for liver lesion:

To evaluate the algorithms' capability to recover low‐contrast structures, we compute the CNR for one liver lesion of the test set (cf. Section 5.5 and Figure C.5, lesion #5). To this end, we place one circular region of interest (ROI) in the lesion and one in the surrounding homogeneous liver tissue. Here we find that all methods improve the CNR compared to the low‐dose reconstruction (Table 5). Remarkably, most methods improve the CNR even compared to the high‐dose reconstruction which is likely due to the pixelwise loss, which smooths the image and thus reduces noise.

**TABLE 5:** Quantitative evaluation of the CNR for one liver metastasis (#5 in Figure C.5).

	CNR	Ranking
HD	2.17	5
LD	0.85	9
CNN‐10 (2017)	2.47	4
RED‐CNN (2017)	**3.13**	1
WGAN‐VGG (2017)	1.52	8
ResNet (2018)	2.93	2
QAE (2019)	1.63	7
DU‐GAN (2021)	1.81	6
TransCT (2021)	2.24	5
Bilateral (2022)	2.74	3

#### CT number accuracies:

**FIGURE 7:**
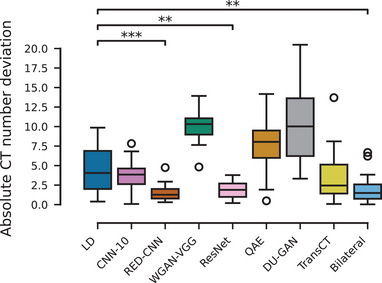
CT number accuracy over 15 ROIs in muscle tissue of chest exams. Statistical significance is indicated with ∗:p<0.05,∗∗:p<0.01,∗∗∗:p<0.001.

We also evaluate the algorithms' ability to recover the CT numbers of the high‐dose reconstruction. To this end, we place five ROIs each for three of the chest exams in muscle tissue and compute the mean CT number for each reconstruction and ROI. We then compute the absolute deviation from the mean CT number of the high‐dose reconstruction and show the results in Figure 7. The mean CT number over all ROIs of the high‐dose scans is 49.76±6.30 HU. We find that three of the eight algorithms (RED‐CNN, ResNet, and Bilateral) perform significantly better than the low‐dose reconstruction in recovering the CT numbers of the high‐dose reconstruction with RED‐CNN achieving the lowest mean deviation with 1.58±1.19 HU. Significance was tested using a Wilcoxon signed‐rank test. The two GAN‐based methods (WGAN‐VGG and DU‐GAN) perform worst in this regard, which can be attributed to the fact that they are not trained exclusively using a pixelwise loss and adversarial losses do not directly enforce gray value consistency.

#### Line profile analysis:

The spatial resolution of an imaging system is commonly evaluated using the MTF. However, since the MTF is not well‐defined for nonlinear algorithms and the task transfer function (TTF) requires phantom measurements, we perform an assessment of the algorithms' ability to recover sharp edges in the image using line profiles. Here we find that while all algorithms reduce the noise compared to the LD reconstruction and stay closer to the high‐dose reconstruction, some algorithms fail to recover sharp edges in the line profile. We provide the line profiles and additional details in Figure D.7 and Appendix D.

## 6 DISCUSSION

In this study, we revisited some of the numerous proposed deep learning‐based algorithms for low‐dose CT image denoising. We discovered several limitations in the experimental setups of these methods that hinder the verifiability of their claimed improvements. To overcome these challenges, we proposed a novel benchmark setup that promotes fair and reproducible evaluations. The setup comprises a unified data preprocessing, rigorous hyperparameter optimization, and evaluation using various metrics, including a novel metric that measures the similarity of radiomic features between the denoised volume and the high‐dose scan.

Upon evaluation of eight deep learning‐based denoising algorithms proposed over the past six years, we find that there has been little progress. Particularly, when evaluated using standard image quality measures such as SSIM and PSNR, we find that no method consistently outperforms one of the earliest methods, RED‐CNN. When evaluated using the radiomic feature similarity, we find that algorithms trained with an adversarial loss significantly outperform methods trained with pixel‐wise losses on some data, indicating that the radiomic feature similarity provides useful information beyond standard, nonclinical image quality metrics. Nonetheless, the newest algorithms considered in our study fail to consistently outperform older ones. An evaluation on lesion annotations and using physical image quality assessment metrics leads to the same conclusion. We also evaluated all methods on subsets of the test data consisting of increasingly difficult slices and find that methods are similarly robust to different amounts of deterioration of the low‐dose scan.

We note that our evaluation mainly focused on distortion (full‐reference) measures^73^ and that the hyperparameter optimization is limited to a single such distortion measure, the SSIM. Future work should consider including more perceptual measures (e.g., based on feature maps of DNNs) both for hyperparameter optimization and subsequent evaluation of the algorithms. This is particularly important, given the recent shift towards using more perceptual loss functions in the field. Other possible extensions include evaluation of more algorithms including score‐based methods^74^, ^75^ and methods that leverage multiple axial slices^74^, ^75^, ^76^, ^77^ as well as training and/or evaluation on more datasets, particularly those that contain lesion annotations.

Similar to “reality checks” in related fields,^78^, ^79^ our study highlights the need for a more rigorous and fair evaluation of novel deep learning‐based denoising methods for low‐dose CT image denoising. We believe that our benchmark setup is a first and important step towards this direction and will help to develop novel and better algorithms.

## CONFLICT OF INTEREST STATEMENT

The authors declare no conflicts of interest.

## Supporting information

Supporting Information

## Footnotes

1
https://github.com/eeulig/ldct‐benchmark

2 We here omit evaluation of RMSE since it is related to the PSNR via $\text{PSNR}=20\log_{10}\left(I_{\max}/\text{RMSE}\right)$, with $I_{\max}$ being the maximum pixel value.

3 This includes features from the following classes (# of features): first order statistics (18), gray level co‐occurrence matrix (24), gray level run length matrix (16), gray level size zone matrix (16), neighboring gray tone difference matrix (4), and gray level dependence matrix (13).

## References

[1] Kalra MK , Maher MM , Toth TL , et al. Strategies for CT radiation dose optimization. Radiology. 2004;230:619‐628. 14739312 10.1148/radiol.2303021726

[2] Brenner DJ , Hall EJ . Computed tomography–an increasing source of radiation exposure. N Engl J Med. 2007;357:2277‐2284. 18046031 10.1056/NEJMra072149

[3] Ziegler A , Koehler T , Proksa R . Noise and resolution in images reconstructed with FBP and OSC algorithms for CT. Med Phys. 2007;34:585‐598. 17388176 10.1118/1.2409481

[4] Sidky EY , Kao C , Pan X . Accurate image reconstruction from few–views and limited–angle data in divergent–beam CT. J X Ray Sci Technol. 2006;14:119‐139.

[5] Balda M , Hornegger J , Heismann B . Ray contribution masks for structure adaptive sinogram filtering. IEEE Trans Med Imaging. 2012;31:1228‐1239. 22333988 10.1109/TMI.2012.2187213

[6] Feruglio PF , Vinegoni C , Gros J , Sbarbati A , Weissleder R . Block matching 3D random noise filtering for absorption optical projection tomography. Phys Med Biol. 2010;55:5401‐5415. 20736500 10.1088/0031-9155/55/18/009 PMC2934766

[7] Li Z , Yu L , Trzasko JD , Lake DS , Blezek DJ , Fletcher JG , McCollough CH , Manduca A . Adaptive nonlocal means filtering based on local noise level for CT denoising. Med Phys. 2014;41:011908. 24387516 10.1118/1.4851635

[8] Manduca A , Yu L , Trzasko JD , et al. Projection space denoising with bilateral filtering and CT noise modeling for dose reduction in CT. Med Phys. 2009;36:4911‐4919. 19994500 10.1118/1.3232004 PMC4108640

[9] Sukovic P , Clinthorne N . Penalized weighted least‐squares image reconstruction for dual energy x‐ray transmission tomography. IEEE Trans Med Imaging. 2000;19:1075‐1081. 11204845 10.1109/42.896783

[10] Chen H , Zhang Y , Zhang W , Liao P , Li K , Zhou J , Wang G . Low‐dose CT denoising with convolutional neural network. In: 2017 IEEE 14th International Symposium on Biomedical Imaging (ISBI 2017) . IEEE; 2017:143‐146.

[11] Chen H , Zhang Y , Zhang W , et al. Low‐dose CT via convolutional neural network. Biomed Opt Express. 2017;8:679‐694. 28270976 10.1364/BOE.8.000679 PMC5330597

[12] Chen H , Zhang Y , Kalra MK , et al. Low‐dose CT with a residual encoder‐decoder convolutional neural network. IEEE Trans Med Imaging. 2017;36:2524‐2535. 28622671 10.1109/TMI.2017.2715284 PMC5727581

[13] Fan F , Shan H , Kalra MK , et al. Quadratic autoencoder (Q‐AE) for low‐dose CT denoising. IEEE Trans Med Imaging. 2020;39:2035‐2050. 31902758 10.1109/TMI.2019.2963248 PMC7376975

[14] Heinrich MP , Stille M , Buzug TM . Residual U‐Net convolutional neural network architecture for low‐dose CT denoising. Curr Dir Biomed Eng. 2018;4:297‐300.

[15] Huang Z , Zhang J , Zhang Y , Shan H . DU‐GAN: generative adversarial networks with dual‐domain U‐Net‐based discriminators for low‐dose CT denoising. IEEE Trans Instrum Meas. 2022;71:1‐12.

[16] Kang E , Min J , Ye JC . A deep convolutional neural network using directional wavelets for low‐dose x‐ray CT reconstruction. Med Phys. 2017;44:e360‐e375. 29027238 10.1002/mp.12344

[17] Ramanathan S , Ramasundaram M . Low dose CT image reconstruction using deep convolutional residual learning network. SN Comput Sci. 2023;4:720.

[18] Shan H , Padole A , Homayounieh F , et al. Competitive performance of a modularized deep neural network compared to commercial algorithms for low‐dose CT image reconstruction. Nat Mach Intell. 2019;1:269‐276. 33244514 10.1038/s42256-019-0057-9 PMC7687920

[19] Wagner F , Thies M , Gu M , et al. Ultralow‐parameter denoising: trainable bilateral filter layers in computed tomography. Med Phys. 2022;49:5107‐5120. 35583171 10.1002/mp.15718

[20] Yang Q , Yan P , Zhang Y , et al. Low‐dose CT image denoising using a generative adversarial network with Wasserstein distance and perceptual loss. IEEE Trans Med Imaging. 2018;37:1348‐1357. 29870364 10.1109/TMI.2018.2827462 PMC6021013

[21] Yang S , Pu Q , Lei C , Zhang Q , Jeon S , Yang X . Low‐dose CT denoising with a high‐level feature refinement and dynamic convolution network. Med Phys. 2023;50:3597‐3611. 36542402 10.1002/mp.16175

[22] Zhang Z , Yu L , Liang X , Zhao W , Xing L . TransCT: dual‐path transformer for low dose computed tomography. In: International Conference on Medical Image Computing and Computer Assisted Intervention . MICCAI; 2021.

[23] Humphries T , Coulter S , Si D , Simms M , Xing R . Comparison of deep learning approaches to low dose CT using low intensity and sparse view data. In: Bosmans H , Chen G‐H , Gilat Schmidt T , eds. Medical Imaging 2019: Physics of Medical Imaging. SPIE; 2019:156.

[24] Ma Y‐J , Ren Y , Feng P , He P , Guo X‐D , Wei B . Sinogram denoising via attention residual dense convolutional neural network for low‐dose computed tomography. Nucl Sci Tech. 2021;32:41.

[25] Yang L , Li Z , Ge R , Zhao J , Si H , Zhang D . Low‐dose CT denoising via sinogram inner‐structure transformer. IEEE Trans Med Imaging. 2023;42:910‐921. 36331637 10.1109/TMI.2022.3219856

[26] Zainulina E , Chernyavskiy A , Dylov DV . No‐reference denoising of low‐dose CT projections. In: 2021 IEEE 18th International Symposium on Biomedical Imaging (ISBI) . IEEE; 2021:77‐81.

[27] Hong Z , Zeng D , Tao X , Ma J . Learning CT projection denoising from adjacent views. Med Phys. 2023;50:1367‐1377. 36414024 10.1002/mp.16115

[28] Cormack AM . Representation of a function by its line integrals, with some radiological applications. J Appl Phys. 1963;34:2722‐2727.

[29] Gordon R , Bender R , Herman GT . Algebraic reconstruction techniques (ART) for three‐dimensional electron microscopy and x‐ray photography. J Theor Biol. 1970;29:471‐481. 5492997 10.1016/0022-5193(70)90109-8

[30] Andersen AH , Kak AC . Simultaneous algebraic reconstruction technique (SART): a superior implementation of the art algorithm. Ultrason Imaging. 1984;6:81‐94. 6548059 10.1177/016173468400600107

[31] Missert AD , Leng S , Yu L , McCollough CH . Noise subtraction for low‐dose CT images using a deep convolutional neural network. In: Proceedings of the Fifth International Conference on Image Formation in X‐Ray Computed Tomography, Salt Lake City, UT, USA . 2018:399‐402.

[32] Baguer DO , Leuschner J , Schmidt M . Computed tomography reconstruction using deep image prior and learned reconstruction methods. Inverse Probl. 2020;36:094004.

[33] Yin X , Zhao Q , Liu J , et al. Domain progressive 3D residual convolution network to improve low‐dose CT imaging. IEEE Trans Med Imaging. 2019;38:2903‐2913. 31107644 10.1109/TMI.2019.2917258

[34] Chao L , Zhang P , Wang Y , Wang Z , Xu W , Li Q . Dual‐domain attention‐guided convolutional neural network for low‐dose cone‐beam computed tomography reconstruction. Knowledge Based Syst. 2022;251:109295.

[35] Zhang Y , Hu D , Zhao Q , et al. CLEAR: comprehensive learning enabled adversarial reconstruction for subtle structure enhanced low‐dose CT imaging. IEEE Trans Med Imaging. 2021;40:3089‐3101. 34270418 10.1109/TMI.2021.3097808

[36] Zhou B , Zhou SK , Duncan JS , Liu C . Limited view tomographic reconstruction using a cascaded residual dense spatial‐channel attention network with projection data fidelity layer. IEEE Trans Med Imaging. 2021;40:1792‐1804. 33729929 10.1109/TMI.2021.3066318 PMC8325575

[37] Zhou B , Chen X , Xie H , Zhou SK , Duncan JS , Liu C . DuDoUFNet: dual‐domain under‐to‐fully‐complete progressive restoration network for simultaneous metal artifact reduction and low‐dose CT reconstruction. IEEE Trans Med Imaging. 2022;41:3587‐3599. 35816532 10.1109/TMI.2022.3189759 PMC9812027

[38] McCollough CH , Bartley AC , Carter RE , et al. Low‐dose CT for the detection and classification of metastatic liver lesions: results of the 2016 low dose CT grand challenge. Med Phys. 2017;44:e339‐e352. 29027235 10.1002/mp.12345 PMC5656004

[39] McCollough C , Chen B , Holmes III DR , et al. Low dose CT image and projection data (data set). The Cancer Imaging Archive. 2020. 10.7937/9NPB-2637

[40] Divel SE , Pelc NJ . Accurate image domain noise insertion in CT images. IEEE Trans Med Imaging. 2020;39:1906‐1916. 31870981 10.1109/TMI.2019.2961837

[41] Horenko I , Pospíšil L , Vecchi E , et al. Low‐cost probabilistic 3D denoising with applications for ultra‐low‐radiation computed tomography. J Imaging. 2022;8:156. 35735955 10.3390/jimaging8060156 PMC9224620

[42] Wang Z , Bovik A , Sheikh H , Simoncelli E . Image quality assessment: from error visibility to structural similarity. IEEE Trans Image Process. 2004;13:600‐612. 15376593 10.1109/tip.2003.819861

[43] Verdun FR , Racine D , Ott JG , et al. Image quality in CT: from physical measurements to model observers. Physica Med. 2015;31:823‐843. 10.1016/j.ejmp.2015.08.007 26459319

[44] Renieblas GP , Nogués AT , González AM , Gómez‐Leon N , Del Castillo EG . Structural similarity index family for image quality assessment in radiological images. J Med Imaging. 2017;4:035501. 10.1117/1.JMI.4.3.035501 PMC5527267 28924574

[45] Ohashi K , Nagatani Y , Yoshigoe M , et al. Applicability evaluation of full‐reference image quality assessment methods for computed tomography images. J Imaging Inform Med. 2023;36:2623‐2634. 10.1007/s10278-023-00875-0 PMC10584745 37550519

[46] Mason A , Rioux J , Clarke SE , et al. Comparison of objective image quality metrics to expert radiologists' scoring of diagnostic quality of MR images. IEEE Trans Med Imaging. 2020;39:1064‐1072. 31535985 10.1109/TMI.2019.2930338

[47] Sheikh H , Bovik A . Image information and visual quality. IEEE Trans Image Process. 2006;15:430‐444. 16479813 10.1109/tip.2005.859378

[48] Pan S , Flores J , Lin CT , Stayman JW , Gang GJ . Generative adversarial networks and radiomics supervision for lung lesion synthesis. Proc SPIE‐Int Soc Opt Eng. 2021;11595:115950O. 10.1117/12.2582151 PMC8516144 34658481

[49] Wei L , Hsu W . Efficient and accurate spatial‐temporal denoising network for low‐dose CT scans. In: Medical Imaging with Deep Learning . 2021.

[50] Patwari M , Gutjahr R , Marcus R , et al. Reducing the risk of hallucinations with interpretable deep learning models for low‐dose CT denoising: comparative performance analysis. Phys Med Biol. 2023;68:19LT01. 10.1088/1361-6560/acfc11 37733068

[51] Barrett HH , Myers KJ . Foundations of Image Science. Wiley; 2004.

[52] Richard S , Husarik DB , Yadava G , Murphy SN , Samei E . Towards task‐based assessment of CT performance: system and object MTF across different reconstruction algorithms. Med Phys. 2012;39:4115‐4122. 22830744 10.1118/1.4725171

[53] Vaishnav JY , Jung WC , Popescu LM , Zeng R , Myers KJ . Objective assessment of image quality and dose reduction in CT iterative reconstruction. Med Phys. 2014;41:071904. 24989382 10.1118/1.4881148

[54] Samei E , Bakalyar D , Boedeker KL , et al. Performance evaluation of computed tomography systems: summary of AAPM Task Group 233. Med Phys. 2019;46:e735‐e756. 31408540 10.1002/mp.13763

[55] Hsieh J , Liu E , Nett B , Tang J , Thibault J‐B , Sahney S . A New Era of Image Reconstruction: TrueFidelity™ . White Paper. GE Healthcare; 2019.

[56] Franzen R . Kodak Lossless True Color Image Suite (data set). 1999. https://r0k.us/graphics/kodak/

[57] Martin D , Fowlkes C , Tal D , Malik J . A database of human segmented natural images and its application to evaluating segmentation algorithms and measuring ecological statistics. In: Proceedings Eighth IEEE International Conference on Computer Vision (ICCV 2001) . Vol 2. IEEE; 2001:416‐423.

[58] Zhang L , Wu X , Buades A , Li X . Color demosaicking by local directional interpolation and nonlocal adaptive thresholding. J Electron Imaging. 2011;20:023016.

[59] Huang J‐B , Singh A , Ahuja N . Single image super‐resolution from transformed self‐exemplars. In: 2015 IEEE Conference on Computer Vision and Pattern Recognition (CVPR) . IEEE; 2015:5197‐5206.

[60] Zhang K , Zuo W , Chen Y , Meng D , Zhang L . Beyond a Gaussian denoiser: residual learning of deep CNN for image denoising. IEEE Trans Image Process. 2017;26:3142‐3155. 28166495 10.1109/TIP.2017.2662206

[61] Bergstra J , Bardenet R , Bengio Y , Kégl B . Algorithms for hyper‐parameter optimization. In: Advances in Neural Information Processing Systems (NeurIPS) . Vol. 24. Curran Associates, Inc.; 2011.

[62] Bergstra J , Bengio Y . Random search for hyper‐parameter optimization. J Mach Learn Res. 2012;13:281‐305.

[63] Snoek J , Larochelle H , Adams RP . Practical Bayesian optimization of machine learning algorithms. In: Advances in Neural Information Processing Systems . Vol. 25. Curran Associates, Inc.; 2012.

[64] Liu C , Gao C , Xia X , Lo D , Grundy J , Yang X . On the reproducibility and replicability of deep learning in software engineering. ACM Trans Softw Eng Methodol. 2021;31:15:1‐15:46.

[65] Kc P , Zeng R , Farhangi MM , Myers KJ . Deep neural networks‐based denoising models for CT imaging and their efficacy. In: Medical Imaging 2021: Physics of Medical Imaging. Vol. 11595. SPIE; 2021:105‐117.

[66] Goodfellow I , Pouget‐Abadie J , Mirza M , et al. Generative adversarial nets. In: Advances in Neural Information Processing Systems . Vol. 27. Curran Associates, Inc.; 2014.

[67] Arjovsky M , Chintala S , Bottou L . Wasserstein generative adversarial networks. In: International Conference on Machine Learning (ICML) . PMLR; 2017:214‐223.

[68] Schonfeld E , Schiele B , Khoreva A . A U‐Net based discriminator for generative adversarial networks. In: IEEE Conference on Computer Vision and Pattern Recognition (CVPR) . IEEE; 2020:8204‐8213.

[69] Wasserthal J , Breit H‐C , Meyer MT , et al. TotalSegmentator: robust segmentation of 104 anatomic structures in CT images. Radiol Artif Intell. 2023;5:e230024. 37795137 10.1148/ryai.230024 PMC10546353

[70] van Griethuysen JJM , Fedorov A , Parmar C , et al. Computational radiomics system to decode the radiographic phenotype. Cancer Res. 2017;77:e104‐e107. 29092951 10.1158/0008-5472.CAN-17-0339 PMC5672828

[71] Mann HB , Whitney DR . On a test of whether one of two random variables is stochastically larger than the other. Ann Math Stat. 1947;18:50‐60.

[72] Zhao H , Gallo O , Frosio I , Kautz J . Loss functions for image restoration with neural networks. IEEE Trans Comput Imaging. 2017;3:47‐57.

[73] Blau Y , Michaeli T . The perception‐distortion tradeoff. In: 2018 IEEE/CVF Conference on Computer Vision and Pattern Recognition . IEEE; 2018:6228‐6237.

[74] Li Q , Li C , Yan C , et al. Ultra‐low dose CT image denoising based on conditional denoising diffusion probabilistic model. In: 2022 International Conference on Cyber‐Enabled Distributed Computing and Knowledge Discovery (CyberC) . 2022:198‐205.

[75] Gao Q , Li Z , Zhang J , Zhang Y , Shan H . CoreDiff: contextual error‐modulated generalized diffusion model for low‐dose CT denoising and generalization. IEEE Trans Med Imaging. 2024;43:745‐759. 37773896 10.1109/TMI.2023.3320812

[76] Zhou Z , Huber NR , Inoue A , McCollough CH , Yu L . Multislice input for 2D and 3D residual convolutional neural network noise reduction in CT. J Med Imaging. 2023;10:014003. 10.1117/1.JMI.10.1.014003 PMC9888548 36743869

[77] Shi J , Elkilany O , Fischer A , Suppes A , Pelt DM , Batenburg KJ . Lodoind: introducing a benchmark low‐dose industrial CT dataset and enhancing denoising with 2.5D deep learning techniques. In: 13th Conference on Industrial Computed Tomography (iCT), Wels Campus, Austria , 2024. 10.58286/29228

[78] Melis G , Dyer C , Blunsom P . On the state of the art of evaluation in neural language models. In: International Conference on Learning Representations . 2018.

[79] Musgrave K , Belongie S , Lim S‐N . A metric learning reality check. In: Vedaldi A , Bischof H , Brox T , Frahm J‐M , eds. Computer Vision – ECCV 2020. Lecture Notes in Computer Science. Springer International Publishing; 2020:681‐699.

