# Supplementary material for: Benchmarking deep learning‐based low‐dose CT image denoising algorithms
Source: Med Phys. 2024 Sep 17;51(12):8776–88. doi: 10.1002/mp.17379 (PMC11656299; doi:10.1002/mp.17379)
Supplement: Supplementary file 1 — Supporting Information [file MP-51-8776-s001.pdf]

Appendix: Benchmarking Deep Learning-Based Low-Dose CT Image Denoising Algorithms

A. Implementation and verification of algorithms

The eight algorithms implemented in this study were all implemented in PyTorch and will be made publicly available together with our benchmark suite. In Fig. A.1 we provide a flowchart of how we implemented and verified a given denoising algorithm.

Implementation For implementation (Fig. A.1; top) of an algorithm we first checked if an implementation was open-sourced by the authors of the original paper, which was the case for the four newest algorithms considered in this study (Tab. A.1). For two of these, implementations were in TensorFlow and had to be translated to PyTorch. For two other algorithms, we found open-source implementations by a third party. Only for two (relatively simple and old) algorithms, namely CNN-10 and ResNet, no implementation was found, and we had to solely rely on the information provided in the original paper for our implementation. For all other algorithms we closely compared results and implementation of individual modules in architecture (*e.g.*, residual blocks) and training pipeline (*e.g.*, loss computation).

Verification For all algorithms we performed unit testing of each module. We then checked whether the authors performed experiments on the *LDCT Image and Projection dataset* or a subset thereof (*e.g.*, *NIHAAPM-Mayo Clinic LDCT Grand Challenge*). If this was the case, we verified that our results (with similar hyperparameters as described by the authors) were quantitatively similar to those reported in the paper. If this was not the case, we had to rely on a qualitative evaluation to verify that our implementation was correct.

B. Usage of the benchmark suite

We provide our benchmark suite as a Python package that can be easily used by researchers to evaluate their algorithms. The package contains the following components:

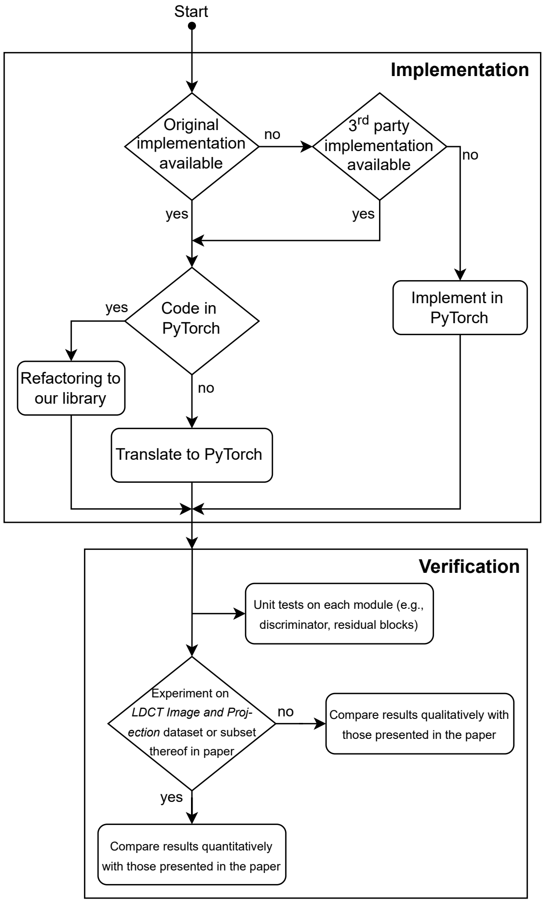

Figure A.1: Flowchart of the implementation and verification process performed for each algorithm considered in this study.

- **Data loader** for the training, validation, and test sets we used of the *LDCT Image and Projection dataset*. This includes preprocessing steps such as normalization and random cropping and makes sure that each algorithm is trained and evaluated using the same data.

- **Base trainer** that provides a base class that provides methods for training, validating and logging of a given algorithm. Researchers can inherit from this class to easily implement their own algorithms in our benchmark suite.

- **Hyperparameter optimization pipeline** which can be used to optimize hyperparameters for a given algorithm using Bayesian optimization. Researchers can provide command line arguments to their algorithm through which hyperparameters are defined. After specifying hyperparameters to be optimized and their priors in a YAML file the pipeline will optimize these arguments for the given algorithm. An example of such a configuration file is shown in Tab. B.2.

Table B.2: Example of a hyperparameter optimization configuration file for CNN-10.

```
method: bayes
metric:
  goal: maximize
  name: SSIM
name: hpopt-cnn10
parameters:
  adam_b1:
    value: 0.9
  adam_b2:
    value: 0.999
  data_norm:
    value: meanstd
  data_subset:
    value: 1.0
  datafolder:
    value: /path/to/LDCTData
  iterations_before_val:
    value: 1000
  lr:
    distribution: log_uniform_values
    max: 0.01
    min: 1.0e-05
  max_iterations:
    distribution: int_uniform
    max: 100000
    min: 1000
  mbs:
    distribution: int_uniform
    max: 128
    min: 2
  num_workers:
    value: 4
  optimizer:
    value: adam
  patchsize:
    distribution: int_uniform
    max: 128
    min: 32
  seed:
    value: 1332
  trainer:
    value: cnn10
```

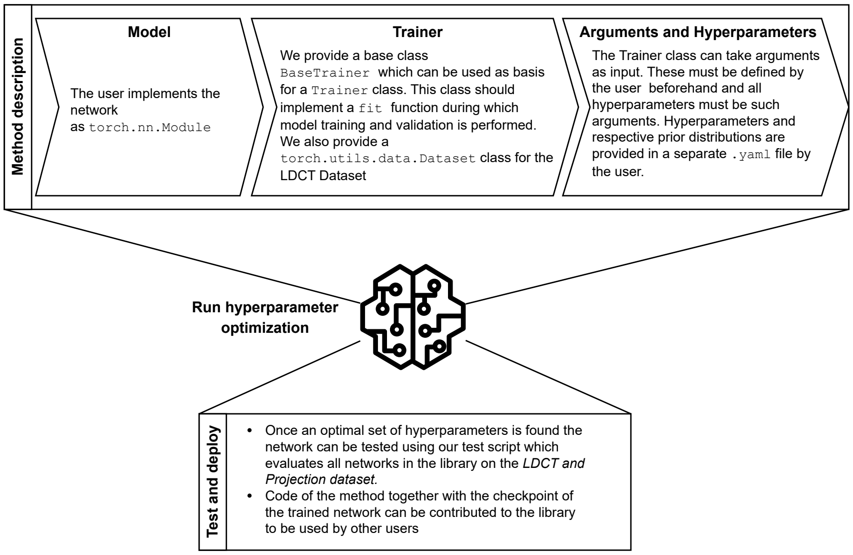

Figure B.2: Flowchart of our benchmark pipeline.

- **Evaluation scripts** to evaluate a given algorithm using the same metrics and settings as in our study. This includes the evaluation of radiomic feature similarity and lesion annotations.

- **Model hub** in which pretrained models of all algorithms considered in this study are provided and to which researchers can contribute their own models.

A flowchart of our benchmark pipeline is shown in Fig. B.2. We provide more information in the documentation of the package.

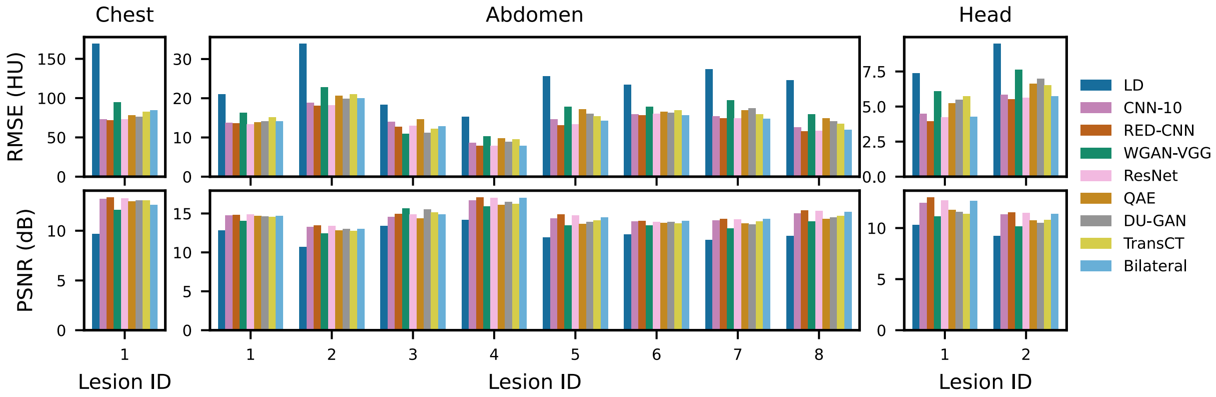

Figure C.3: Quantitative evaluation for each of the lesions in the test set. Lesion IDs correspond to # provided in Figs. C.4 to C.6.

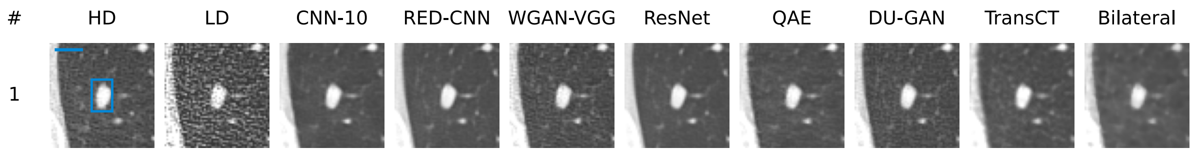

Figure C.4: Solid, non-calcified lung nodule on chest exam from the test set. Blue line indicates one centimeter, blue bounding-box indicates the lesion annotation.

C. Evaluation on lesions

We show reconstruction results on all lesions and algorithms in Figs. C.4 to C.6 and present quantitative evaluation of all individual lesions in Fig. C.3. The quantitative evaluation is performed inside the bounding box annotation shown in blue in Figs. C.4 to C.6.

D. Line profile analysis

For the chest exam (Fig. D.7a) and abdomen exam (Fig. D.7b), we observe that all algorithms reduce the noise compared to the LD reconstruction (blue curve; top-left line plot) and stay closer to the high dose reconstruction (black curve in all plots). However, some algorithms fail to recover sharp edges in the line profile (CNN-10, QAE, TransCT, and Bilateral for the chest exam and Bilateral for the abdomen exam). We find that RED-CNN, ResNet, WGAN-VGG, and DU-GAN perform best in this regard for both exams. For the head exam (Fig. D.7c), while all algorithms reduce noise compared to the LD reconstruction, they all fail to recover the sharp edges in the line profile except TransCT.

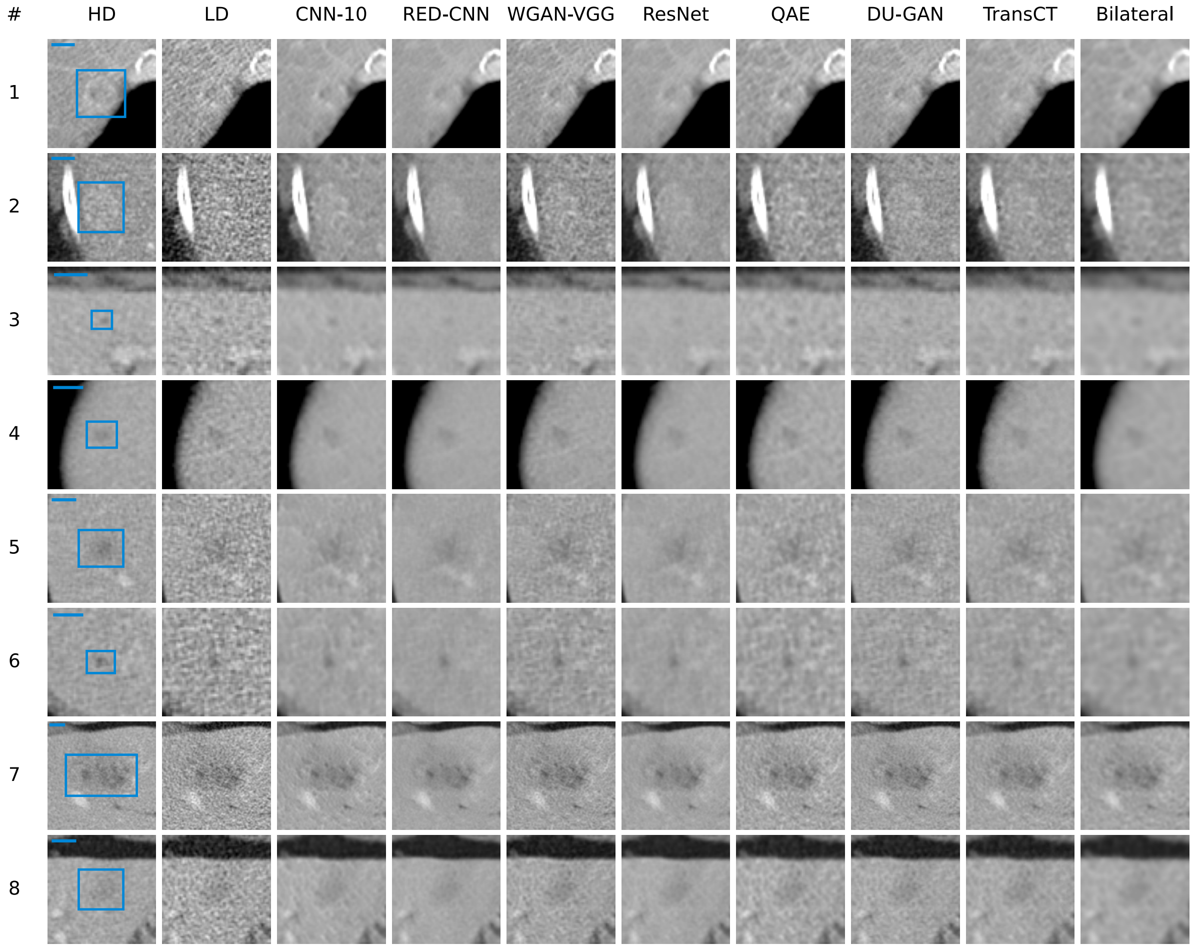

Figure C.5: Pathologies on abdomen exams from the test set. Blue line indicates one centimeter, blue bounding-box indicates the lesion annotation. (1, 2, 5, 7, 8): metastasis, (3, 6): cyst, 4: hemangioma.

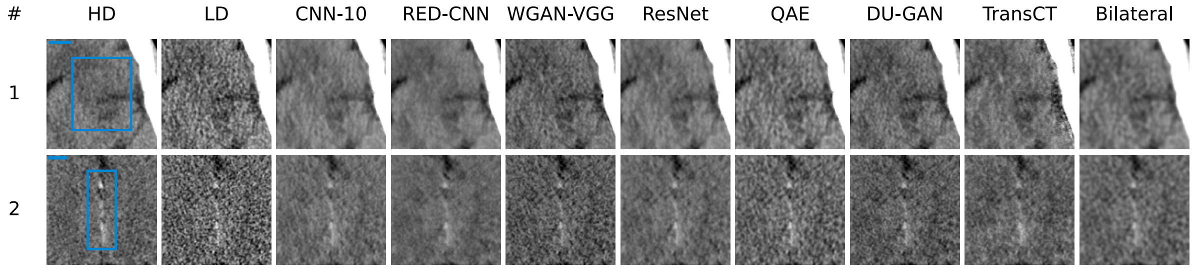

Figure C.6: Pathologies on head exams from the test set. Blue line indicates one centimeter, blue bounding-box indicates the lesion annotation. 1: acute frontal infarct, 2: traumatic subarachnoid hemorrhage.

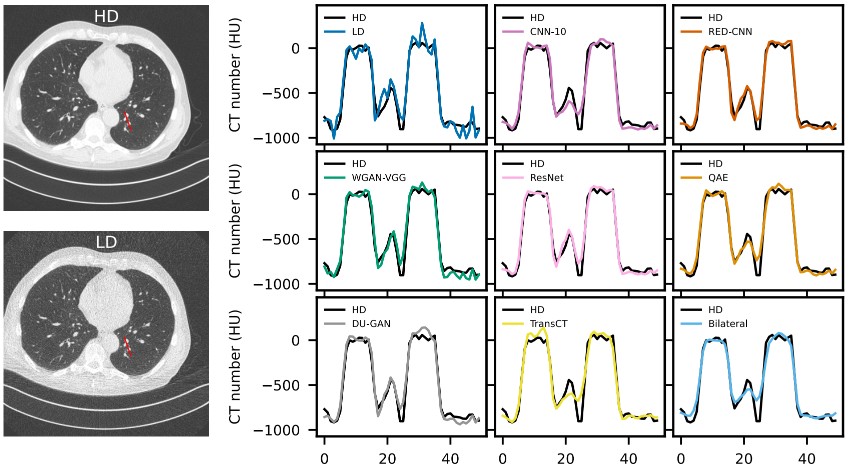

(a) Line profile for chest exam

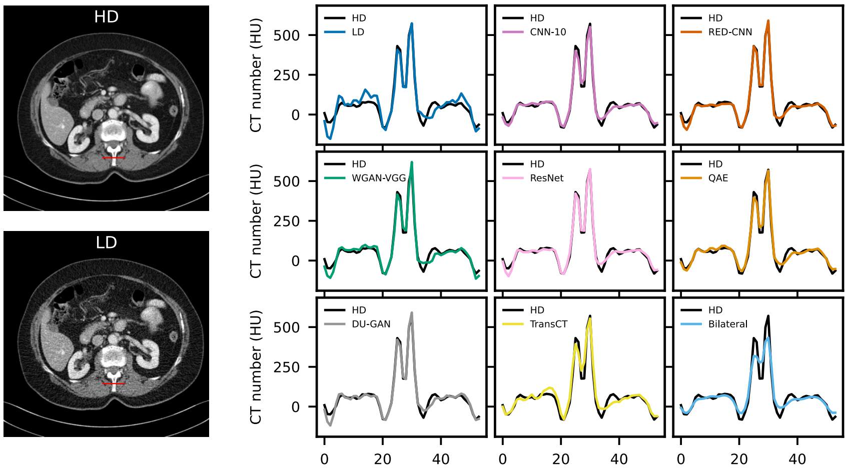

(b) Line profile for abdomen exam

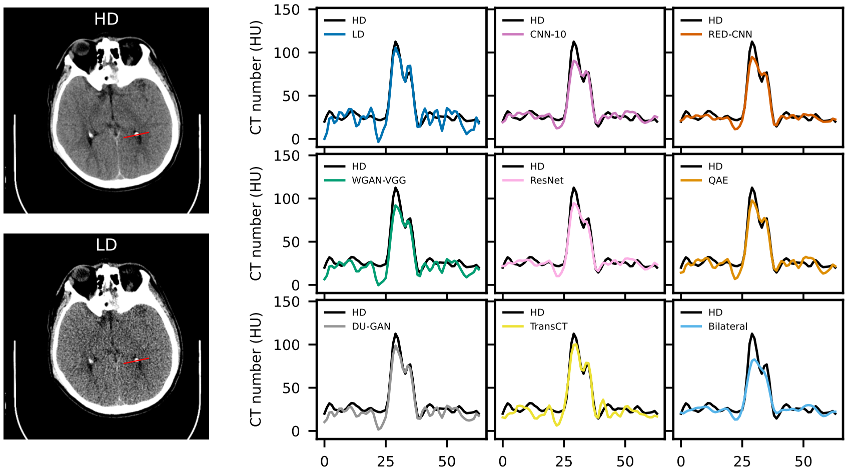

(c) Line profile for head exam

Figure D.7: Line profiles for chest (a), abdomen (b), and head (c) exams. The line along which the profile is computed is indicated in red in the high dose and low dose image.
